# Diverticulitis: a comprehensive review with usual and unusual complications

**DOI:** 10.1007/s13244-016-0532-3

**Published:** 2016-11-22

**Authors:** Mehmet Ruhi Onur, Erhan Akpinar, Ali Devrim Karaosmanoglu, Cavid Isayev, Musturay Karcaaltincaba

**Affiliations:** 1 Department of Radiology, Hacettepe University, School of Medicine, Ankara, Turkey; 2 Hacettepe Universitesi Tıp Fakültesi Hastanesi, 06100 Sıhhiye, Ankara Turkey

**Keywords:** Diverticulitis, Complication, Pylephlebitis, Ultrasonography, Computed tomography

## Abstract

**Abstract:**

Diverticulitis is characterized by inflammation of the outpouchings of the bowel wall. Imaging findings of diverticulitis include edematous thickening of the bowel wall with inflammatory changes within the adjacent mesenteric fat. Uncomplicated diverticulitis can be treated conservatively; however, complicated diverticulitis may not be responsive to medical treatment and life-threatening conditions may occur. In this review, we aimed to illustrate the ultrasonography (US) and computed tomography (CT) features of diverticulitis and its complications including perforation, phlegmon, abscess, ascending septic thrombophlebitis (phylephlebitis), bleeding, intestinal obstruction, and fistula.

***Teaching Points*:**

• *Complications of diverticulitis may be highly variable.*

• *It may be difficult to diagnose diverticulitis as underlying cause of severe complications.*

• *MDCT is essential for the primary diagnosis of the acute diverticulitis and its complications.*

## Introduction

Diverticulitis is one of the most frequent bowel emergencies presenting with acute abdomen. Acute diverticulitis constitutes 3.8 % of causes of abdominal pain in patients presented to the emergency departments [1]. Approximately 10 %–25 % of patients with known colonic diverticulosis will have diverticulitis in their lifetime [2]. The underlying pathophysiology of diverticulitis is the obstruction of the diverticular ostium by a stool fragment or food particles and subsequent inflammation.

Ultrasonography (US) is generally the first imaging modality used in the evaluation of acute abdomen. In diverticulitis, US demonstrates inflamed diverticulum as a noncompressible outpouching of a bowel wall with thickened and hypoechoic wall often containing an obstructive fecalith at the ostium. Adjacent bowel wall edema and thickening with edematous hyperechoic mesentery can be visualized on US [3]. Gentle compression with US transducers generally induce tenderness and pain. Computed tomography is the mainstay imaging technique in the diagnosis of diverticulitis and its complications. Severity of inflammation, involvement of bowel segment and local and distant complications of diverticulitis can be assessed with CT.

Complications of diverticulitis may be highly variable, and it may be difficult to diagnose diverticulitis as an underlying cause of severe complications. Small sized, well-contained perforations are common in the course of the disease and most cases can be managed conservatively with antibiotics and supportive medical treatment. However, unusual and more severe complications such as non-contained perforation, phlegmon and abscess, phylephlebitis, intestinal obstruction, bleeding, and fistula necessitate intensive management. These conditions should be promptly diagnosed and treated in order to prevent increased morbidity and mortality.

## Complications of diverticulitis

### Perforation

**Fig. 1:**
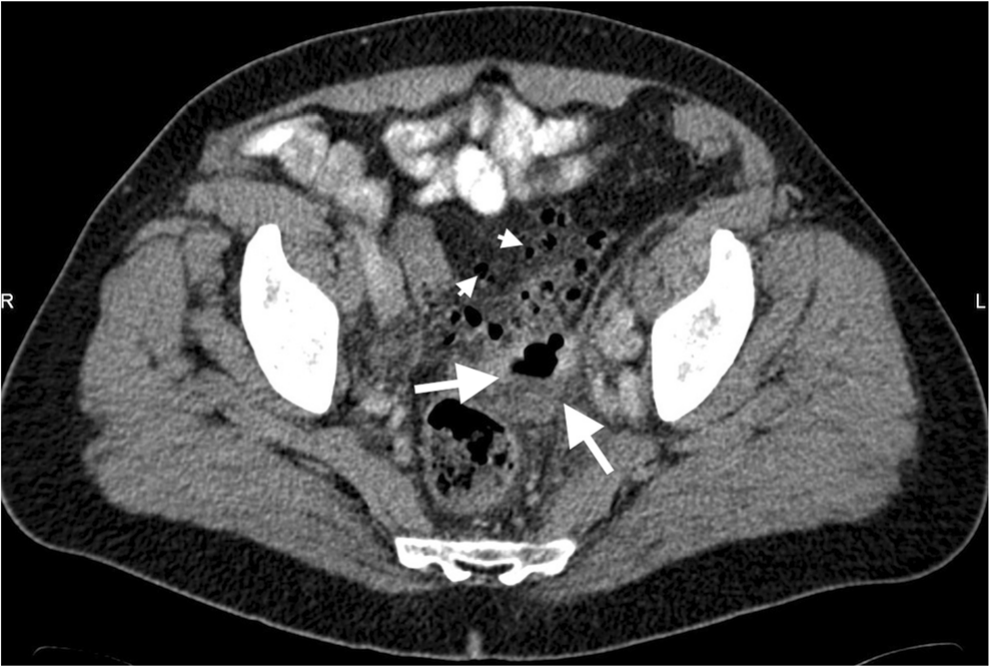
Thirty-eight year-old male with diverticulitis and well-contained perforation. Axial contrast-enhanced CT demonstrates edema and thickening of the sigmoid colon wall with multiple diverticulums. A fluid collection adjacent to the sigmoid colon (*arrows*) is an abscess caused by perforation of the diverticulitis. Free air pockets (*arrowhead*) confined to the pericolonic region are seen

**Fig. 2:**
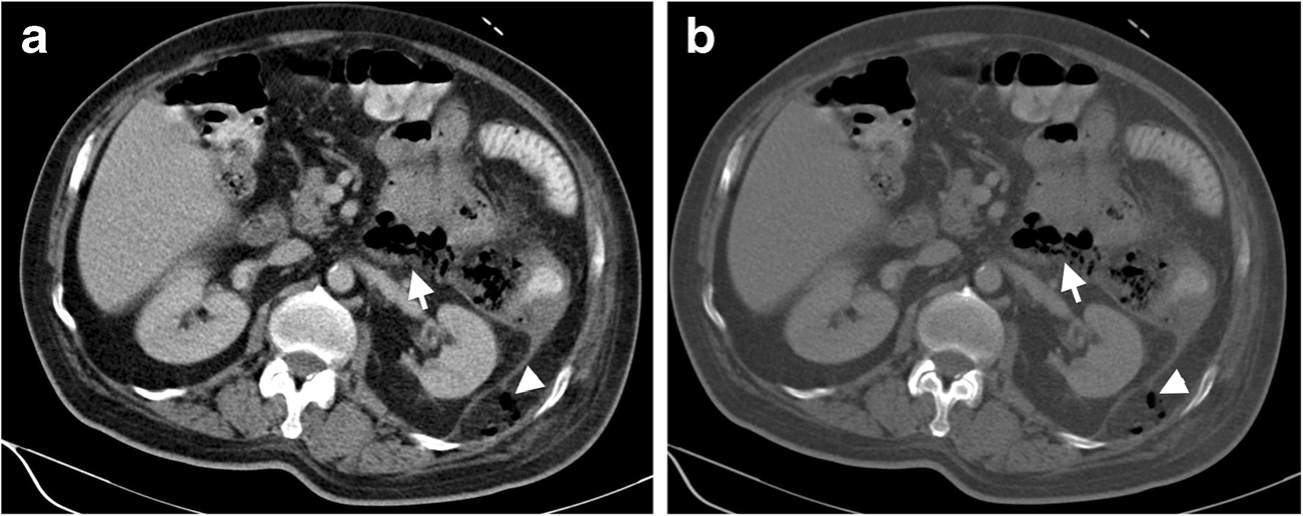
Seventy-two-year-old female with free perforation complicating diverticulitis. Axial contrast enhanced CT images at parenchymal window (**a**) and bone window (**b**) demonstrate a ‘dirty mass’ (*arrows*) formed by stool nearby sigmoid colon diverticulitis. Free air (*arrowheads*) and inflammatory fat stranding resulting from perforation of diverticulitis are seen posterior to the pararenal fascia

Perforation of diverticulitis occurs secondary to severe inflammation of bowel wall layers with subsequent necrosis and loss of intestinal wall integrity. Perforation from colonic diverticulitis almost always occurs on the left side [3]. Well-contained perforations manifest as small and self-limited; however, non-contained perforations which occur in 1 %–2 % of patients with acute diverticulitis may lead to local abscess and fistula formation [4–6] (Fig. 1). Free air is usually detected locally with well-contained perforation while widespread intraabdominal free air is detected in large non-contained perforations [4, 5] (Fig. 2). Intraperitoneal perforation may present with acute abdominal pain, nausea and vomiting. Retroperitoneal air can result from perforation of second and third portions of duodenum, posterior aspect of the ascending, descending and sigmoid colon segments. The clinical presentation may be insidious and relatively silent in these patients causing delayed diagnosis and potentially life-threatening complications.

In the setting of perforated diverticulitis, subdiaphragmatic free air may be seen on upright abdominal X-rays. Sonographic evaluation of perforated diverticulitis performed with a high frequency linear transducer better reveals wall thickening and edema in the affected bowel segment. Diverticular perforation can be challenging to detect sonographically. Multidetector computed tomography (MDCT) is much better suited for detection of free air with a reported success rate around 85 % [7, 8]. Direct signs of perforation on MDCT include focal bowel wall discontinuity, extraluminal gas, and extraluminal enteric contrast agent leakage. Segmental bowel wall thickening, abnormal bowel wall enhancement, perivisceral fat stranding and abscess formation may be detected as indirect findings of perforation [9]. CT images at lung window settings can be helpful in the detection of small air bubbles between bowel segments. Endovascular air bubbles can be seen in mesenteric veins and portal vein in advanced cases. Poor overall medical condition, steroid use and patients experiencing their first diverticulitis attack are more prone to perforation and subsequent peritonitis [10].

Free air resulting from perforation of diverticulitis may be detected in the retroperitoneum and mediastinum and very rarely, in the scrotal cavity [11]. It was proposed that free air may gain access to the mediastinum through the posterior perirenal space and the diaphragmatic hiatus [12].

### Abscess

**Fig. 3:**
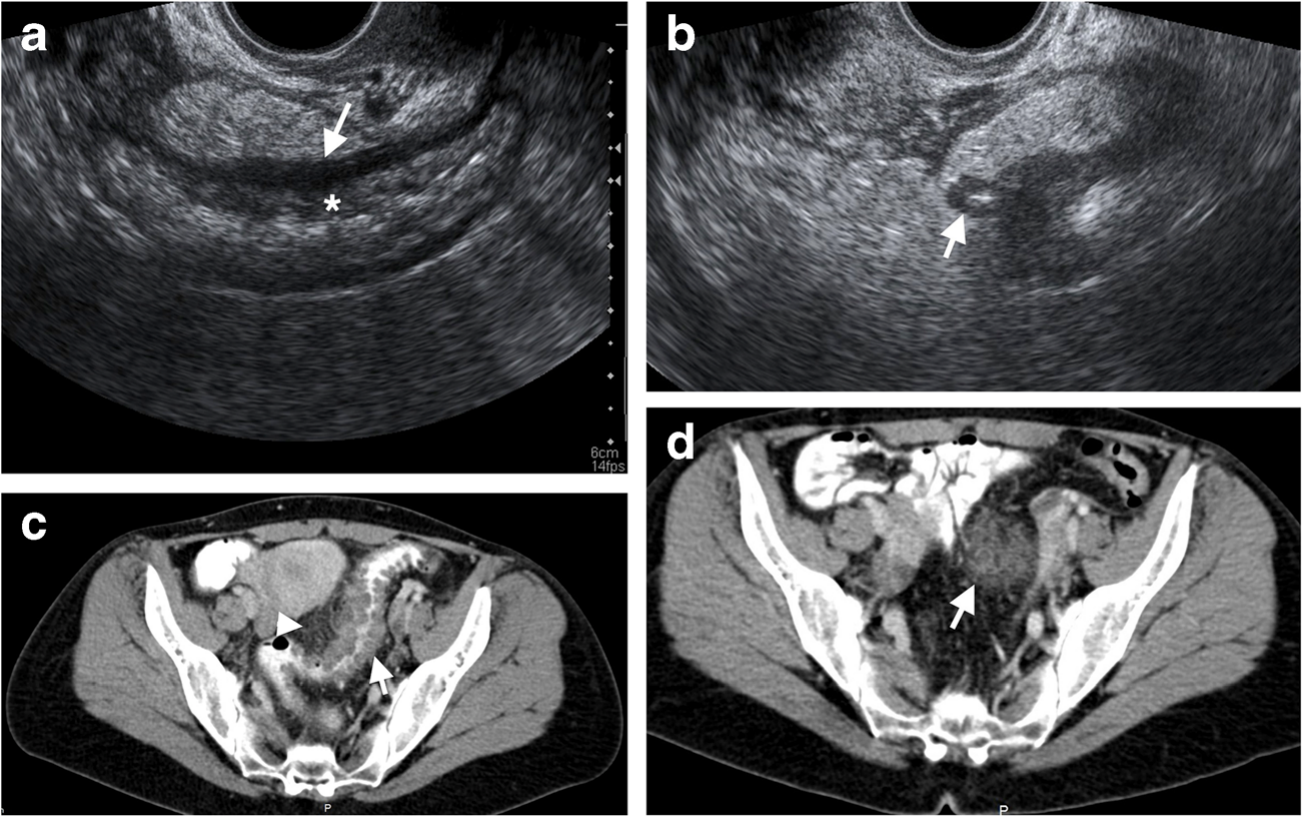
Phlegmon in a 38-year-old female with diverticulitis. **a** Endovaginal US reveals diffuse thickening and edema of the rectum and sigmoid colon mucosa (*) and muscular wall (*arrow*). **b** A diverticulum (*arrow*) with wall thickening, hyperechoic content (fecalith) and surrounding edematous fat tissue represents diverticulitis. **c** Axial contrast-enhanced CT of the same patient demonstrates multiple diverticulum with wall edema in distal sigmoid colon (*arrow*) and pericolonic fat stranding (*arrowheads*) representing diverticulitis. **d** Axial contrast-enhanced CT image at the superior level reveals a phlegmon with a hyperattenuating appearance (*arrow*) compared to adjacent pelvic fat

**Fig. 4:**
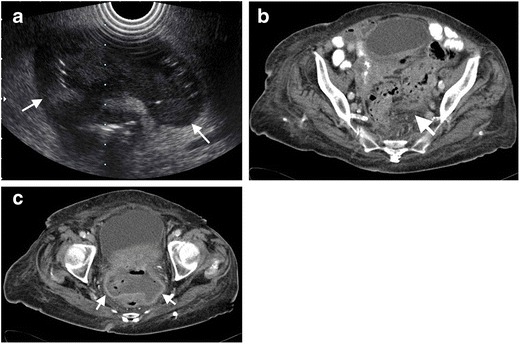
Abscess resulting from a diverticulitis in an 85-year-old female. **a** Endovaginal US demonstrates a fluid collection with gas content and wall thickening (*arrows*) representing abscess. **b** Axial contrast-enhanced CT image reveals diverticulitis with edematous sigmoid colon, multiple diverticulum and surrounding fat stranding (*arrow*). **c** Axial contrast-enhanced CT of the same patient demonstrates a pelvic abscess (*arrows*) with enhancing wall and air content in a Douglas pouch

Diverticulitis may result in phlegmon and abscess formation. Phlegmon is detected as an inflammatory mass with heterogenous contrast enhancement adjacent to diverticulitis while abscess typically manifests as a loculated fluid collection containing air (Figs. 3 and 4). An avidly enhancing wall is another characteristic feature of an abscess. Abscesses may be detected in up to 30 % of cases with acute diverticulitis [13]. On ultrasonography, abscesses appear as hypoechoic fluid collection with an echogenic thick wall. CT is the most commonly utilized modality in the assessment of abscesses and phlegmon. CT provides invaluable information regarding the size and location and as well as providing a roadmap for image guided interventions. Phlegmon appears as a round or ovular shaped high-attenuated mass compared to mesenteric fat on CT (Fig. 3). Epiploic appendagitis as an inflammatory and ischemic condition of appendices epiploica that mimic phlegmon-forming diverticulitis on CT with the appearance of pericolonic fat stranding. Contrast-enhanced CT demonstrates a central focal area of hyperattenuation that corresponds to the thrombosed vein within the inflamed appendage and a thin-high density rim around the inflamed fat in epiploic appendagitis. The wall of the colon adjacent to the epiploic appendagitis is most often normal in thickness [14]. In epiploic appendagitis, inflammation is localized on the anti-mesenteric border of the colon while diverticulitis usually cause inflammation in the mesocolon [15].

Treatment approach to the abscess depends on its size as well as the location and the overall medical condition of the patient. Conservative management may be preferred in abscesses less than 3 cm in size whereas percutaneous drainage or surgical intervention may be required for larger lesions [16]. Complicated diverticulitis are classified according to the Hinchey classification (Table 1). Stage III and IV diverticulitis in the Hinchey classification requires emergency operative treatment.

**Table 1:** Staging complicated diverticulitis by CT

Stage	Modified Hinchey classification	CT findings
0	Mild clinical diverticulitis	Diverticulum ± colonic wall thickening
Ia	Confined pericolonic inflammation/phlegmon	Colonic wall thickening with pericolonic soft tissue changes
Ib	Pericolonic/mesocolic abscess	Ia changes + pericolonic/mesocolic abscess
II	Pelvic, distant intraabdominal or retroperitoneal abscess	Ia changes + distant abscess (generally deep in the pelvis or in interloop regions)
III	Generalized purulent peritonitis	Free gas associated with localized or generalized ascites and possible peritoneal wall thickening
IV	Generalized fecal peritonitis	Same findings as III

**Fig. 5:**
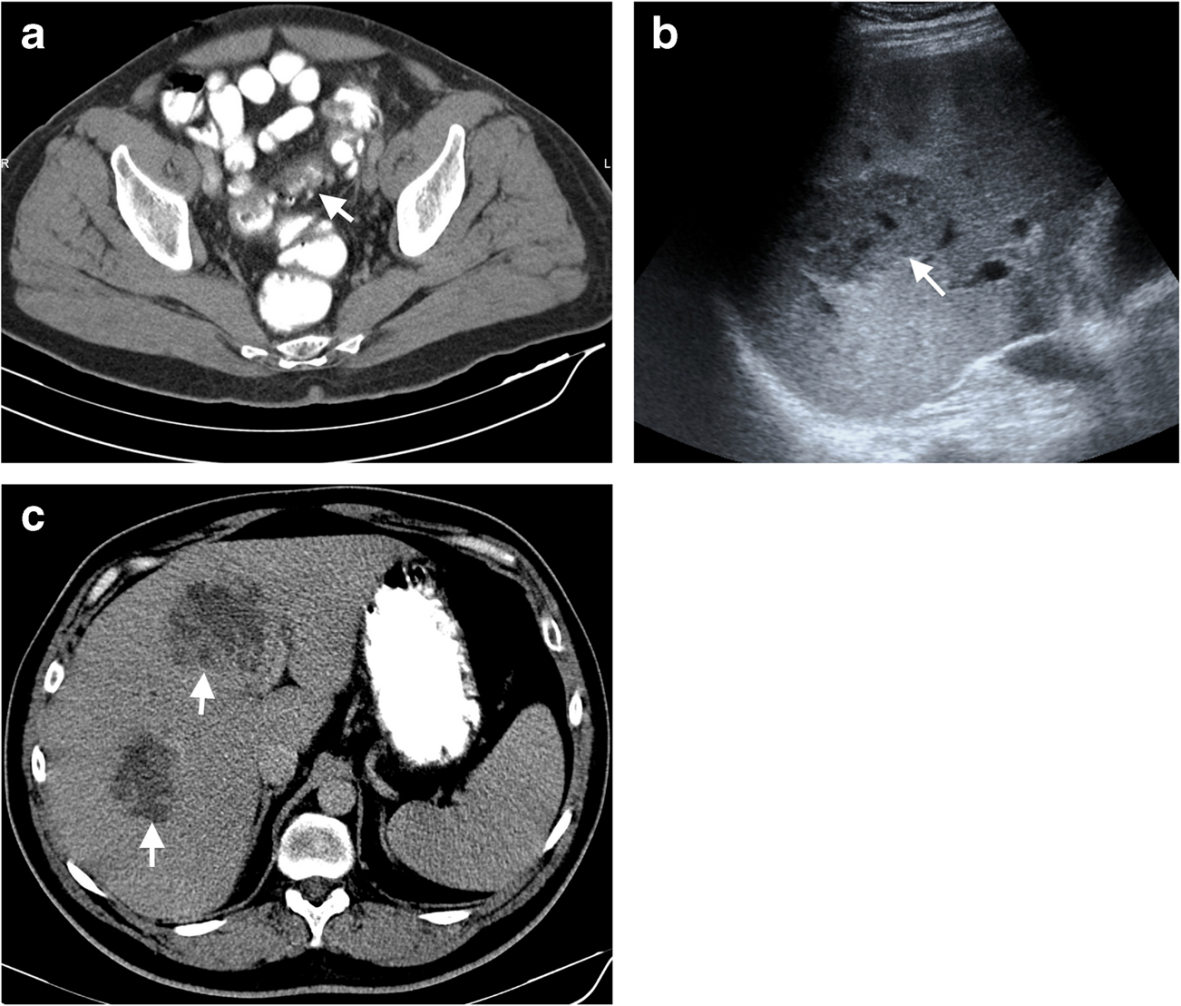
Liver abscess in a 48-year-old male after diverticulitis. **a** Axial CT demonstrates diverticulitis (*arrow*) in the sigmoid colon. **b** Liver abscess secondary to diverticulitis. Gray-scale US demonstrates a liver abscess with a hypoechoic, solid mass appearance (*arrow*) in the liver parenchyma. **c** Liver abscesses manifest with low-attenuating, ill-defined appearance (*arrows*) in the liver parenchyma on axial contrast-enhanced CT

Abscess secondary to diverticulitis may also occur at distant sites such as liver, adnexa, lung and rare localizations such as brain and spine. The liver is the most common remote site of abscess formation [17]. Hematogeneous spread of microorganisms may be due to bacterial invasion into the portal system via colonic mucosal defects [18]. The mortality rate in case of pylephlebitis and liver abscess formation can be as high as 32 % [19]. On US, these hepatic abscesses may appear as highly echogenic fluid collection with thick walls. However, it should be kept in mind that liver abscesses may also mimic an infiltrative solid mass with ill-defined contours on US (Fig. 5). CT is the most commonly used imaging modality and abscesses appear as hypodense fluid-attenuated lesions with intensely enhancing walls and may contain air. Multiphasic imaging (with arterial phase) may aid in differentiating an abscess from possibly other malignant lesions [9].

A tuboovarian abscess may complicate acute diverticulitis of a sigmoid colon due to its close proximity to the adnexa [17]. Sonographic assessment may be of higher yield when performed via the transrectal or transvaginal route. With this approach, the abscess and its anatomic close relationship with the diverticulitis can be precisely outlined. The diagnosis may be challenging with CT as tuboovarian abscesses commonly manifest as a complex multiloculated adnexal mass. The concomitant thickening of the sigmoid mesocolon with or without inflamed sigmoid diverticulum favors accompanying acute colonic diverticulitis in patients presenting with tuboovarian abscess.

### Pylephlebitis

Pylephlebitis, also called ascending septic thrombophlebitis, is a rare complication of intraabdominal infections. It is typically characterized by infective suppurative thrombosis of either the portal vein or its branches, or both [20]. Diverticulitis is the most common underlying cause (30 %) of septic thrombophlebitis of the mesenteric and portal venous system [21]. Other underlying causes of pylephlebitis include appendicitis, necrotizing pancreatitis, bowel perforation, pelvic infection and inflammatory bowel disease [20, 22]. Thrombosis of the superior mesenteric vein is the most common form detected in 42 % of patients, followed by portal vein (39 %), and finally, the inferior mesenteric vein (IMV) (2 %) [18]. Bacteroides fragilis and Escherichia coli are the most common causative organisms in these patients [20].

**Fig. 6:**
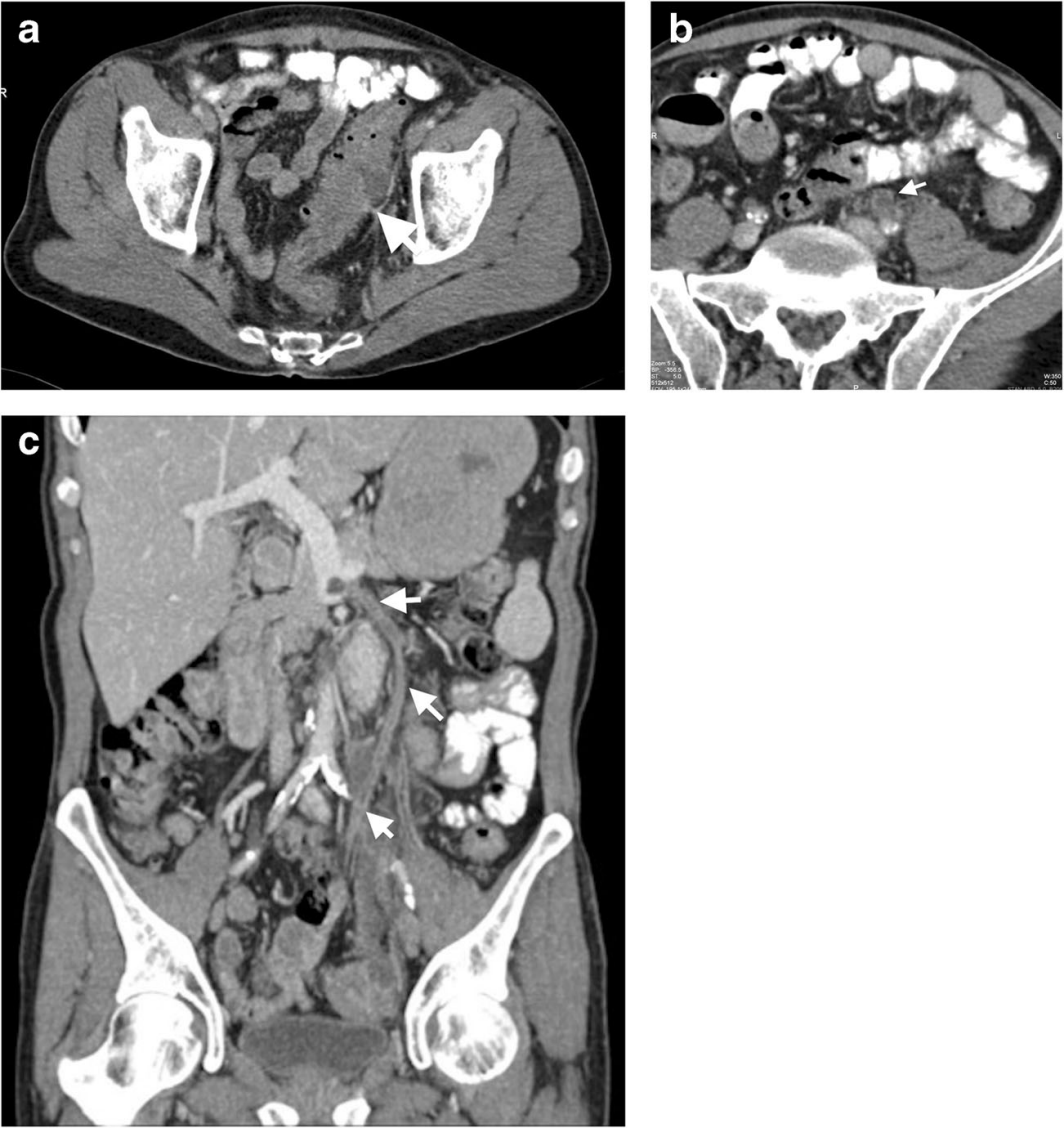
Sixty-year-old male presenting with diverticulitis and IMV thrombosis. **a** Axial contrast-enhanced CT image demonstrates thickening of sigmoid colon wall (*arrow*) with multiple diverticulum. **b** Axial and coronal **c** contrast-enhanced CT images reveal thrombus in the IMV (*arrows*) with adjacent fat stranding representing thrombophlebitis

The involved mesenteric veins in pylephlebitis are closely related with the affected colonic segment. Sigmoid diverticulitis results in thrombosis of the local sigmoid vein with subsequent propagation along the IMV and the portal vein. This process is called ascending thrombophlebitis (Fig. 6) [22].

**Fig. 7:**
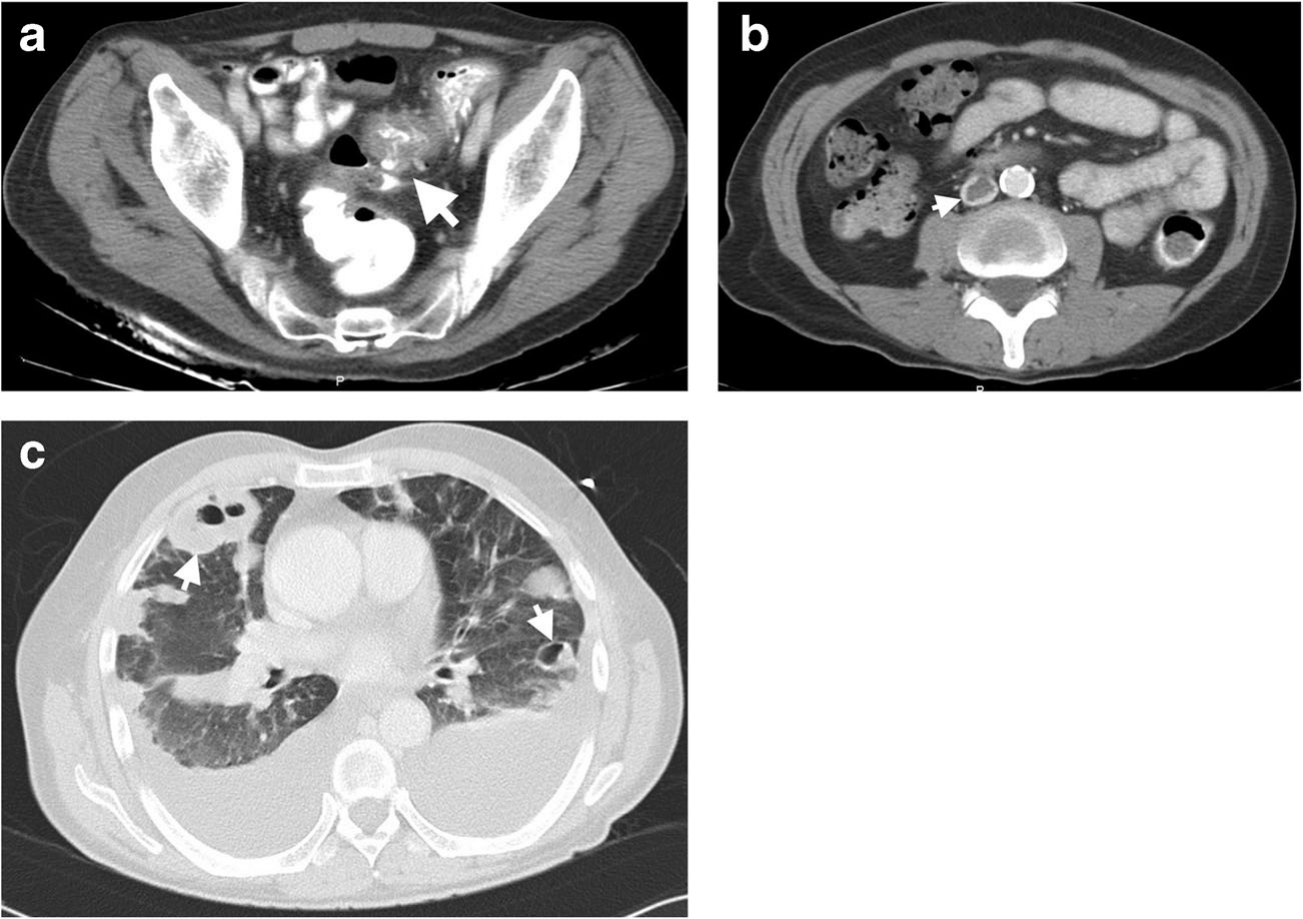
Pulmonary septic emboli in a 58-year-old male resulting from diverticulitis. **a** Axial contrast-enhanced CT of a 58 year-old man demonstrates edema and thickening of the sigmoid colon wall (*arrow*) with diverticulum and adjacent fat stranding. **b** Axial contrast-enhanced CT reveals thrombosis (*arrow*) in the inferior vena cava lumen. **c** Axial contrast-enhanced CT image at lung window setting shows multiple cavitary lesions (*arrows*) with thick wall representing septic emboli

The clinical presentation of septic thrombophlebitis is insidious with vague symptoms, delaying early diagnosis and treatment. Therefore, a high index of clinical suspicion and appropriate use of imaging studies is of critical importance for timely diagnosis. CT is generally the first utilized modality in patients with diverticulitis with pylephlebitis. The diagnosis is generally straightforward with direct visualization of the endoluminal thrombus as a filling defect in the contrast filled mesenteric veins. In the case of portal vein thrombosis, central or peripheral hypoattenuating areas in the liver may be detected as a sign of abnormal hepatic perfusion [22]. For the correct diagnosis with CT, the use of an appropriate CT protocol is mandatory. Several abdominal CT protocols have been proposed, including the “biphasic injection of iodinated contrast” (60 mL at a rate of 2 mL/sec, 30 s of pause followed by a second injection of 60 mL at a rate of 3 mL/sec and then 20 mL of saline) that simultaneously, and nicely, depicts both the arterial and venous structures of the mesentery [22]. Coronal and sagittal reformatted images, in addition to axial images, should be carefully searched for endoluminal thrombus for accurate diagnosis. Curved planar reformatted images may be necessary to assess small caliber vessels such as the IMV. Septic thrombosis in the inferior vena cava may also be seen after diverticulitis, which may result in septic pulmonary emboli and, subsequent, cavitary pulmonary nodules [23] (Fig. 7). Early diagnosis and treatment is important to prevent the formation of embolic abscess formation in distant organs. Appropriate antibiotic use with concomitant anticoagulation are mainstays for the treatment.

### Bowel obstruction

**Fig. 8:**
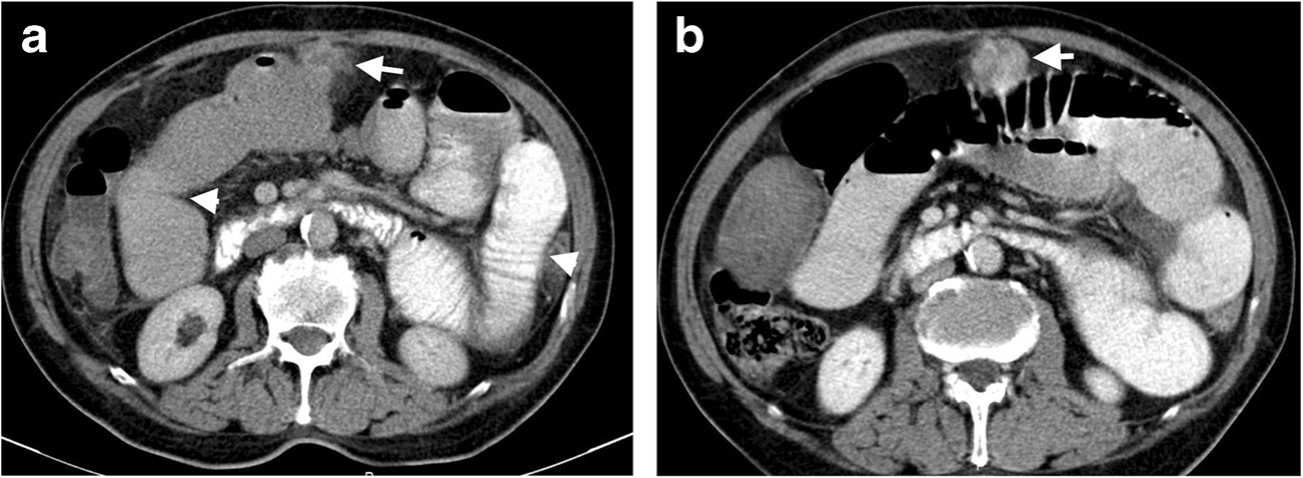
Intestinal obstruction in a 79-year-old male secondary to diverticulitis. **a** Axial contrast-enhanced CT of a 75-year-old man presenting with abdominal pain and distention reveals a diverticulitis with the appearance of a inflamed diverticulum and pericolonic fat stranding (*arrow*) in the transverse colon. Bowel segments proximal to the localization of diverticulitis is dilated. **b** Axial CT performed 2-months after the first CT reveals thickening of the colonic wall (*arrow*) leading to intestinal obstruction

Severe intestinal obstruction in patients with diverticulitis is rare; however, partial obstruction secondary to wall edema and peripheral inflammation or abscess formation may occur. Intramuscular fibrosis seen in the chronic phase may also lead to obstruction in 10–20 % of the cases [24]. In these cases, irregular wall thickening with upstream bowel dilatation is the most common finding (Fig. 8). The main differential diagnosis in cases of acute diverticulitis is an obstructive malignant mass in the colon. While involvement of a long colonic segment (>10 cm) suggests diverticulitis, it should also be kept in mind that both neoplasm and acute diverticulitis may involve a short bowel segment. The most helpful finding of diverticulitis is the detection of a diverticulum in the involved segment; however, colon cancer cannot be confidently excluded based on this finding, as diverticulosis without any active inflammation is also highly prevalent in the general population [16]. Eccentric wall thickening is more common in colonic carcinoma, while concentric wall thickening is more suggestive of acute diverticulitis. Perilesional mesenteric lymph nodes with a short axis diameter exceeding 10 mm are more common in colon cancer than acute diverticulitis [9].

Rectal diverticulitis is a very rare form of diverticulitis and may present as a rectal mass with surrounding inflammatory changes and obstructive findings. Jejunal diverticulitis, also rare, typically presents with adhesion of the epiploic band, resulting in subsequent internal hernia.

Chronic diverticulitis is a variant of diverticulitis that is characterized by persisting symptoms such as abdominal pain for 6 months to 1 year and secondary obstructive symptoms. Intestinal obstruction may develop due to chronic inflammatory changes and associated dense fibrosis [25]. Sigmoid colon is the most commonly affected colonic segment in this form [26]. Barium studies demonstrate narrowing of the involved segment, tethered and speculated folds, and tapered margins with diverticula. Circumferential narrowing of involved segment results from chronic inflammation and fibrosis of the colonic wall and surrounding pericolic fat [25].

### Bleeding

Lower gastrointestinal hemorrhage can be seen in up to 5 % of patients with colonic diverticulosis [27]. Since the outpouchings, representing the diverticula, occur mainly where the vessels pierce the muscularis layer of the colonic wall, both non-complicated diverticulosis and diverticulitis have a tendency to bleed [28]. Bleeding from diverticulitis commonly occurs in chronic diverticulitis with reported prevalence of 17 % [29].

**Fig. 9:**
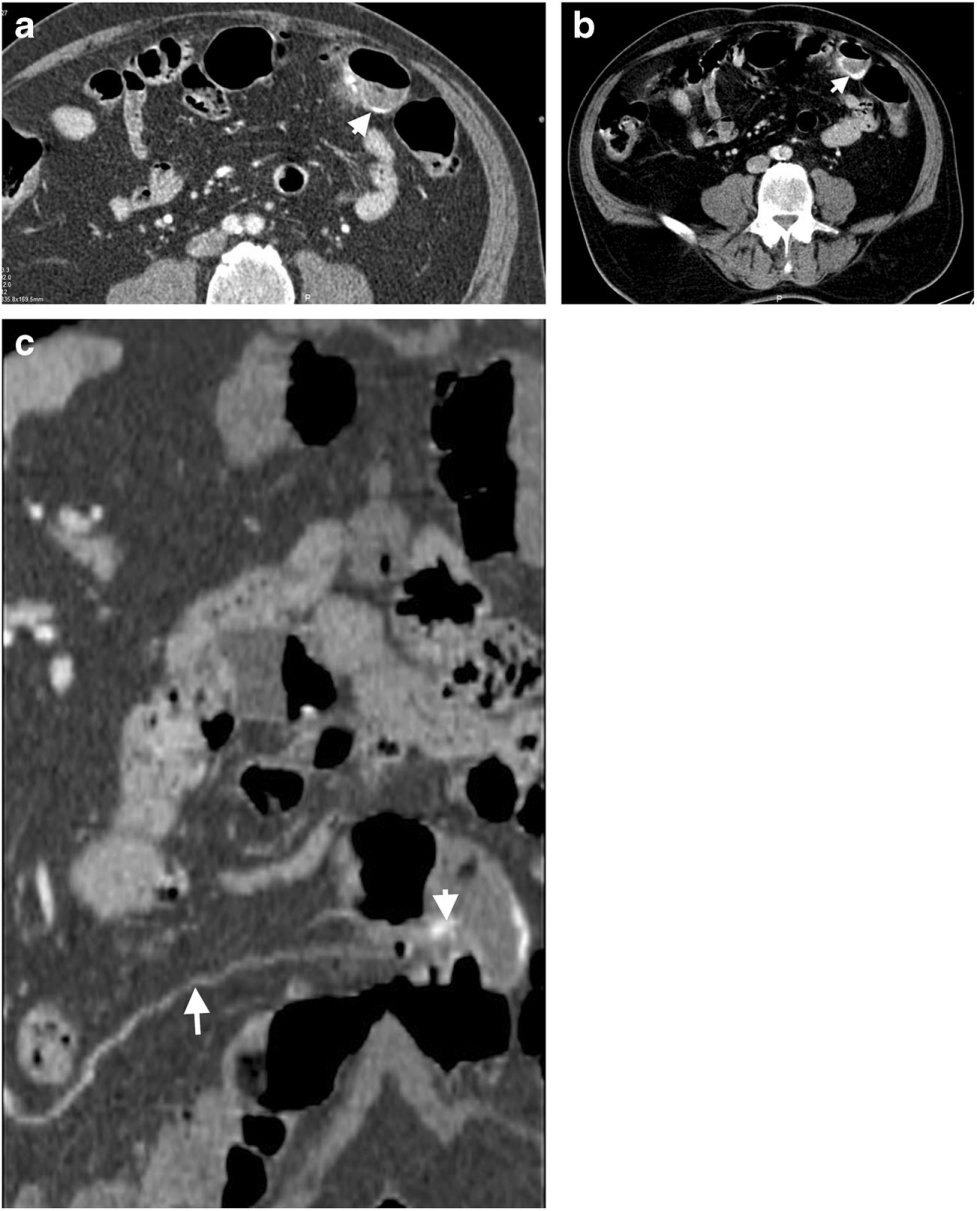
Bleeding diverticulitis in a 70-year-old male. **a** Axial contrast-enhanced CT of a 70-year-old man without oral contrast agent administration reveals intraluminal high attenuating contrast agent leakage (*arrowhead*) in transverse colon. **b** Axial contrast- enhanced CT at venous phase demonstrates increased contrast amount in the lumen (*arrow*) indicating active hemorrhage. **c** Oblique reformatted CT image reveals feeding artery (*arrow*) of the diverticulitis and contrast extravasation (*arrowhead*)

Oral contrast should not be used in patients with a clinical suspicion of gastrointestinal bleeding on CT as it may obscure the active contrast extravasation from the eroded vessel. Bleeding from colonic diverticulitis may be detected on unenhanced CT examinations as hyperdense endoluminal bowel content. Contrast-enhanced CT images in the arterial phase can demonstrate active extravascular contrast extravasation into the diverticulum and bowel lumen, if the flow rate of the bleeding is profuse enough. In these cases progressive contrast pooling in the bowel lumen is another confirmatory sign of active bleeding from the diverticulitis (Fig. 9).

### Fistula

The rate of fistula formation is around 14 % after an episode of acute diverticulitis [9]. They occur when a diverticular abscess breaches the wall integrity of the adjacent anatomic structure [3]. Urinary bladder, ureter, other adjacent intestinal segments, gallbladder, uterus, fallopian tubes, vagina, skin, and the perianal region may all be involved in fistula formation after diverticulitis. Fistula from diverticulitis can be seen as, in the order of decreasing frequency, colovesical, coloenteric and colouterine forms [29].

**Fig. 10:**
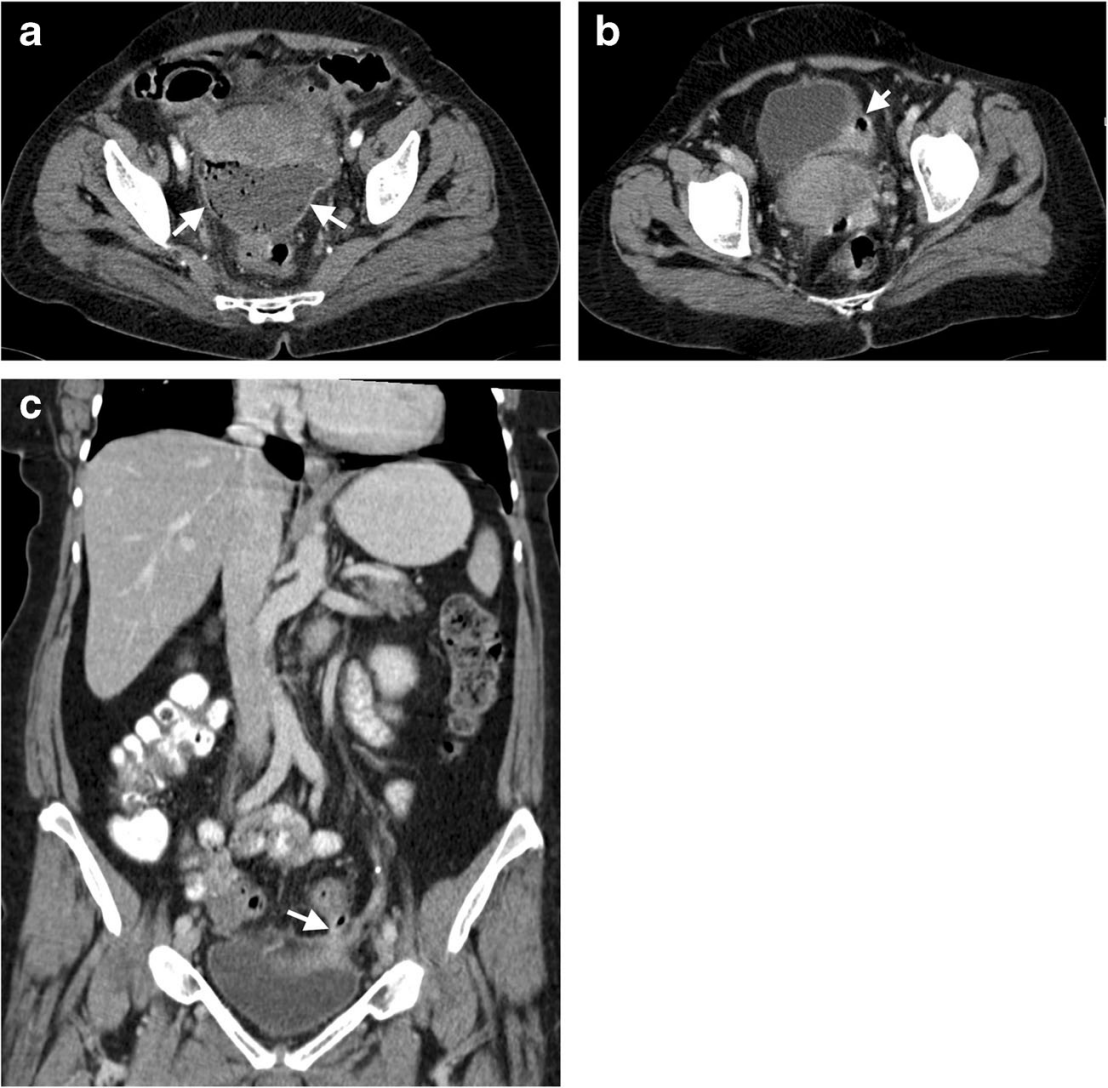
Colovesical fistula in a 46-year-old male after diverticulitis. **a** Axial contrast-enhanced CT of a 62-year-old woman reveals a fluid collection (*arrows*) in the Douglas pouch with air content and peripherally enhancing wall. **b** Contrast-enhanced CT reveals thickening of left bladder wall (*arrow*) adjacent to the diverticulum. **c** Coronal contrast-enhanced CT performed 5 months after first CT demonstrates a fistula (*arrow*) between the sigmoid colon and the bladder

Colovesical fistula present with free air in the bladder with thickening of the adjacent bladder wall [30]. Several episodes of treatment-resistant lower urinary tract infection and the presence of stool or air in the urine are the common symptoms in these patients. Administration of rectal contrast may be helpful for outlining the exact trajectory of the fistula tract. These fistula are most commonly located in the left posterior portion of the bladder, which is in close anatomic location with the sigmoid colon [28] (Fig. 10). Colovesical fistula secondary to diverticulitis differ from those seen in Crohn’s disease. In patients with Crohn’s disease, the fistula occurs generally between the terminal ileum and right anterior surface of the bladder [28].

Colouterine fistula may manifest with myometrial abscess formation [31]. CT may demonstrate air bubbles within the uterine cavity, which is a highly specific finding, if detected. MRI and sonohysterography were also reported to be helpful in detecting colouterine fistula [32, 33].

## Conclusions

Besides the usual signs and symptoms, in the course of the diverticular disease, several unusual complications may be seen. Pylephlebitis, perforation, intestinal obstruction, abscess and fistula formation may be counted among the unusual complications. MDCT is a very robust imaging tool for the primary diagnosis of the acute diverticulitis and its usual and unusual complications. Although CT is the most commonly used modality, radiologists should be familiar with US findings as sonography is generally the first preferred imaging study in the emergency department. Radiologists should be cognizant of the complex unusual complications of acute diverticulitis as they may require a multi-disciplinary treatment approach.
